# Endometriosis in Carriers of a Pathogenic Variant in *BRCA1* or *BRCA2*: A Descriptive Analysis of a Large Multicentral *BRCA* Carrier Cohort

**DOI:** 10.3390/curroncol32120675

**Published:** 2025-12-01

**Authors:** Aghaghia Mokhber, Brynne Stewart, Kathryn L. Terry, Jacek Gronwald, Cezary Cybulski, Raymond H. Kim, Beth Y. Karlan, Louise Bordeleau, Teresa Ramón y Cajal, Tuya Pal, Andrea Eisen, Fergus J. Couch, Dana Zakalik, Nadine Tung, Robert Fruscio, William D. Foulkes, Amber M. Aeilts, Ping Sun, Jan Lubiński, Steven Narod, Joanne Kotsopoulos

**Affiliations:** 1 Women’s College Research Institute, Women’s College Hospital, Toronto, ON M5S 1B2, Canada; 2 Queen’s School of Medicine, Queen’s University, Kingston, ON K7L 3N6, Canada; 3 Temerty Faculty of Medicine, University of Toronto, Toronto, ON M5S 1A1, Canada; 4 Harvard T.H. Chan School of Public Health, Harvard University, Boston, MA 02115, USA; 5 Department of Genetics and Pathology, International Hereditary Cancer Center, Pomeranian Medical University, 70-204 Szczecin, Polandjan.lubinski@pum.edu.pl (J.L.); 6 Princess Margaret Cancer Centre, University Health Network, Toronto, ON M5G 2C4, Canada; 7 Department of Obstetrics and Gynecology, David Geffen School of Medicine, University of California, Los Angeles, CA 90095, USA; 8 Department of Oncology, Juravinski Cancer Centre, McMaster University, Hamilton, ON L8S 4L8, Canada; 9 Department of Medical Oncology, Hospital de la Santa Creu i Sant Pau, 08025 Barcelona, Spain; 10 Vanderbilt-Ingram Cancer Center, Department of Medicine, Vanderbilt University Medical Center, Nashville, TN 37232, USA; 11 Department of Medical Oncology, Sunnybrook Odette Cancer Center, University of Toronto, Toronto, ON M5S 1A1, Canada; 12 Division of Experimental Pathology and Laboratory Medicine, Department of Laboratory Medicine and Pathology, Mayo Clinic, Rochester, MN 55901, USA; 13 Cancer Genetics Program, Beaumont Hospital, Royal Oak, MI 48073, USA; 14 Beth Israel Deaconess Medical Center, Boston, MA 02215, USA; 15 Department of Medicine and Surgery, University of Milan-Bicocca, 20126 Milan, Italy; 16 UO Gynecology, Fondazione IRCCS San Gerardo dei Tintori, 20900 Monza, Italy; 17 McGill Program in Cancer Genetics, Department of Oncology, McGill University, Montreal, QC H3A 0G4, Canada; 18 Division of Human Genetics, Comprehensive Cancer Center, The Ohio State University Medical Center, Columbus, OH 43202, USA; 19 Dalla Lana School of Public Health, University of Toronto, Toronto, ON M5S 1A1, Canada

**Keywords:** *BRCA* mutations, endometriosis, ovarian cancer, prevalence, reproductive epidemiology

## Abstract

**Background:** Endometriosis affects an estimated 10% of reproductive-aged women and is associated with increased ovarian cancer risk. While *BRCA1/2* mutations are established risk factors for ovarian cancer, their association with endometriosis remains unclear. This study aimed to characterize the prevalence and clinical features of endometriosis within a large cohort of *BRCA* mutation carriers. **Methods:** A descriptive analysis was conducted using data from a multi-center longitudinal cohort of women with pathogenic BRCA variants. Reproductive history and related factors were collected through self-reported questionnaires and compared. **Results:** Among 16,950 *BRCA* carriers, the prevalence of endometriosis was 2.4%. Compared to *BRCA* carriers without endometriosis, those with endometriosis were more likely to carry a *BRCA2* mutation, have post-secondary education, and experience earlier menarche. *BRCA* carriers with endometriosis had a lower ovarian cancer prevalence than those without (10% vs. 15%, *p* < 0.001). **Conclusions:** This is the first study of this scale to report the prevalence of endometriosis among *BRCA* mutation carriers, which was lower than previously reported in the general population. The association between endometriosis and ovarian cancer does not appear to be generalizable to this population. Further prospective studies are warranted to clarify this association among *BRCA* mutation carriers.

## Simple Summary

Endometriosis is a common condition that affects women during their reproductive years and is linked to chronic pain and fertility challenges. Some studies suggest that endometriosis may also increase the risk of ovarian cancer. Women who carry *BRCA* gene mutations already face a higher risk of ovarian cancer, but it is unclear whether they are also more likely to have endometriosis. In this large study of nearly 17,000 women with *BRCA* mutations, endometriosis was found to be much less common than in the general population, and it did not appear to increase ovarian cancer risk. These findings suggest that the connection between endometriosis and ovarian cancer may differ for women with *BRCA* mutations. Understanding these differences could help guide future research and inform personalized cancer prevention and reproductive health strategies for women at genetic risk.

## 1. Introduction

Endometriosis is a chronic gynecological disorder characterized by the presence of endometrial-like tissue outside the uterus, affecting approximately 10% of reproductive-aged women globally [1,2]. This multifactorial condition is associated with significant morbidity, notably chronic pelvic pain, dysmenorrhea, dyspareunia, infertility, and depression [1,2,3,4,5,6,7]. The pathophysiology of endometriosis is complex with potential contributions from retrograde menstruation, immune dysregulation, hormonal imbalances, and genetic predisposition [8,9,10,11]. Endometriosis is estrogen-dependent, with estrogen driving lesion growth and progesterone resistance limiting its suppression [10,11]. This condition has been associated with increased risk of ovarian cancer, particularly the clear cell and endometrioid subtypes [12,13], with some recent evidence also suggesting a modest link with high-grade serous subtypes [14].

Although twin studies have suggested a heritable component to endometriosis, no high-penetrance susceptibility genes have been identified [15,16]. Findings from genome-wide association studies suggest a role of genetic variation in *WNT4*, *FN1*, and *GREB1* genes, all of which are implicated in estrogen signaling and inflammation [1,17]. *BRCA1* and *BRCA2* are tumor suppressor genes that play a critical role in maintaining genomic stability, among other critical functions. Pathogenic (or likely pathogenic) *BRCA* variants confer the highest known lifetime risks of developing ovarian and/or fallopian tube cancer [18].

Given the association between endometriosis and ovarian cancer, it is important to understand how endometriosis presents in women at high genetic risk of ovarian cancer, including *BRCA* carriers. While *BRCA* mutations are well-established in hereditary cancer syndromes, their relationship to benign gynecologic conditions such as endometriosis is not evaluated. Moreover, other burdens of endometriosis, including chronic pain, infertility, and mental health impacts, remain underexplored in this high-risk population. This study aims to evaluate the prevalence and clinical characteristics of endometriosis in a large multicenter cohort of *BRCA* carriers and to provide real-world insights into how endometriosis manifests in this high-risk population.

## 2. Methods

### 2.1. Study Population

Participants were identified from a longitudinal study of women aged 18 to 70 with a confirmed pathogenic or likely pathogenic variant in the *BRCA1* or *BRCA2* gene (carriers hereafter) and has been previously described in detail [19]. Briefly, eligible participants had undergone genetic testing based on a personal or family history of breast and/or ovarian cancer. While various methods were used to detect mutations, all identified variants were confirmed through direct DNA sequencing. Informed written consent was obtained from all participants. The study was conducted in accordance with the ethical standards of the relevant institutional or national research committees and with the 1964 Declaration of Helsinki and its later amendments. Ethical approval was obtained from the Institutional Review Boards of all participating centers.

### 2.2. Data Collection

Participants complete a research questionnaire at the time enrollment (between 1995 and 2024), which was administered either in person during a clinic visit or remotely via telephone or mail. The questionnaire gathered self-reported data on established and potential risk factors for breast and ovarian cancer, including personal and family cancer history, medical and reproductive history, medication use, and select lifestyle factors. Menopausal status was also assessed, with questions addressing natural, surgical, medication-induced, or other causes of menopause. Follow-up questionnaires were distributed every two years to collect updated information on exposures, new cancer diagnoses, treatments received, and vital status. These follow-ups were conducted by mail or telephone by a research assistant.

Self-reported diagnoses of primary invasive ovarian or fallopian tube cancer, collectively referred to as ovarian cancer, were collected. The questionnaires asked specifically about a personal diagnosis of endometriosis (yes/no), including details on the data of diagnosis and treatments received. We also included cases of endometriosis identified incidentally during surgery. Date of diagnosis was based on the participant’s report where available.

### 2.3. Eligible Participants

All *BRCA1* and *BRCA2* mutation carriers who completed at least one follow-up questionnaire were eligible for inclusion. Participants were excluded if they reported a history of any malignancy other than breast or ovarian cancer (n = 2301). To limit misclassification, women who had undergone bilateral salpingo-oophorectomy (BSO) or hysterectomy prior to an endometriosis diagnosis were also excluded (n = 20). For analyses involving ovarian cancer, self-reported ovarian cancer diagnoses were included only if they occurred after the reported endometriosis diagnosis and in women without a history of prophylactic BSO. Women with missing data on endometriosis status or date of diagnosis were excluded from all analyses (n = 263). After applying these criteria, 16,950 women remained eligible: 402 with self-reported endometriosis and 16,547 without.

### 2.4. Statistical Analysis

Descriptive statistics were used to summarize baseline characteristics, comparing *BRCA* carriers with and without endometriosis. Continuous variables were presented as means and corresponding minimum and maximum values (min–max) and compared using Student’s *t*-test, while categorical variables were presented as counts and percentages and compared using the χ^2^ test. All statistical analyses were conducted using SAS On Demand for Academics (Cary, NC, USA), and statistical significance was set at *p* ≤ 0.05.

## 3. Results

Among the 16,950 *BRCA* carriers included in the study, 403 (2.4%) had a self-reported diagnosis of endometriosis (Table 1). The mean age at diagnosis of endometriosis was 35.2 years (min–max: 13–72). The mean follow-up time for the cohort was 5.5 years (min–max: 2–26), with a longer average follow-up among women with endometriosis (8.1 years; min–max: 2–24) compared with those without endometriosis (5.5 years; min–max: 2–26). A greater proportion of participants with endometriosis had post-secondary education compared with those without endometriosis (75% vs. 67%). Women with endometriosis reported an earlier age at menarche (mean: 12.7 vs. 13.0 years) and were more likely to be postmenopausal at the time of data collection (85% vs. 72%). Among postmenopausal women, those with endometriosis experienced menopause at a younger age (41.5 vs. 44.3 years), with surgical menopause being more common (69% vs. 55%). Parity also differed significantly, with a higher proportion of women with endometriosis never having given birth (29% vs. 20%). Women with endometriosis were more likely to have a history of oral contraceptive (75% vs. 64%) and HRT use (31% vs. 20%). Rates of hysterectomy and BSO were also higher in women with endometriosis (53% vs. 32% and 68% vs. 45%, respectively).

Participants with endometriosis were more likely to carry a *BRCA2* mutation compared with those without endometriosis (34% vs. 28%, *p* = 0.012) (Table 2). The prevalence of ovarian cancer was lower in women with endometriosis (10% vs. 15%, *p* < 0.01).

## 4. Discussion

In this report of endometriosis in a large cohort of *BRCA* carriers, we observed a lower prevalence of endometriosis (2.4%) compared to that estimated from women in the general population (~10%) [1,2]. In addition, the prevalence of ovarian cancer was significantly lower in *BRCA* carriers with endometriosis compared to those without (10% vs. 15%). Although limited by small number of endometriosis cases and self-reported diagnosis, these findings suggest that *BRCA* carriers with endometriosis are not at an increased risk of ovarian cancer.

Despite the large number of *BRCA* carriers in our study, a very small proportion ever reported a history of endometriosis. Table 3 summarizes the small but growing body of literature that has investigated the prevalence of endometriosis among *BRCA* mutation carriers, with sample sizes ranging from 37 to 4926 participants [20,21,22,23,24,25,26]. Reported prevalence among *BRCA* carriers ranged from 1.6% to 34.2%, with some studies focusing solely on *BRCA* mutation carriers and others comparing carriers to non-carriers or women with other genetic mutations. Among studies that only analyzed *BRCA* mutation carriers, endometriosis prevalence was generally low, with rates under 5% in all cohorts [24,26]. While one study reported higher rates of endometriosis in *BRCA* carriers than in mutation-negative women [22], others found lower rates among carriers relative to non-carriers [20,23]. Among the case-only studies, which focused on cancer patients, prevalence rates were closer to the documented prevalence of endometriosis in the general population [1]. Rates ranged from approximately 8% to 10% across studies, with no significant differences between *BRCA* carriers and non-carriers [21,25,26]. Lower prevalence of endometriosis in this population may be explained by the high rates of prophylactic surgeries (BSO, Hysterectomy) among *BRCA* carriers [27,28,29,30], which remove the primary sites of endometriotic lesions and reduce the likelihood of a formal endometriosis diagnosis. Another potential factor is diagnostic bias due to cancer-focused medical surveillance [27], where clinical attention in *BRCA* carriers is directed toward malignancy risk rather than benign gynecologic conditions. As a result, endometriosis may be underdiagnosed or underreported in this population.

Despite this lower prevalence, several well-established risk factors for endometriosis, including earlier menarche [31,32,33], lower early adulthood body mass [34,35], and nulliparity [33,36], were observed in our *BRCA* carrier cohort. These findings suggest that *BRCA* mutation status does not necessarily alter traditional risk profiles for endometriosis, reinforcing that hormonal and metabolic influences continue to play a role in disease development even within this high-risk genetic population. A particularly notable finding of our study was the lower prevalence of ovarian cancer among *BRCA* carriers with endometriosis compared to those without (10% vs. 15%, *p ≤* 0.001), a contrast to the general population, where endometriosis is associated with an increased risk of ovarian cancer [12,13,14]. Our findings are aligned with previous reports on the association between endometriosis and ovarian cancer risk among *BRCA* mutation carriers. Although limited by sample size, Gersekowski et al. reported a weaker association between endometriosis and ovarian cancer in *BRCA* mutation carriers compared to non-carriers (IRR = 0.75, 95% CI: 0.54–1.01) [25]. Similarly, none of the endometriosis patients with a *BRCA* mutation were found to have cancer in a study conducted by Loizzi et al. [26], while 81.8% of cancers associated with endometriosis in another study were associated with endometrioid carcinoma over other gynecological malignancies [21]. These findings may reflect differences in tumor histology [12,14,37]. Endometriosis has been most strongly linked to clear cell ovarian cancer [12], with emerging evidence suggesting a modest association with high-grade serous carcinomas (HGSC) [14]. In contrast, *BRCA*-driven ovarian cancers are predominantly HGSC [37]. Since endometriosis is not strongly linked to HGSC development, its impact on cancer risk may be less pronounced in *BRCA* carriers. It is also possible that, in *BRCA*-associated ovarian cancer, endometriosis may represent an incidental rather than causative finding, consistent with emerging histologic distinctions between endometriosis-correlated and endometriosis-incidental ovarian cancer [38]. Additionally, the low prevalence of ovarian cancer among *BRCA* carriers with endometriosis could be influenced by early risk-reducing surgeries [27,28,29,30], including bilateral oophorectomy which would dramatically reduce ovarian cancer risk. Socioeconomic and healthcare access factors may have also influenced the findings. Women with endometriosis in our *BRCA* cohort were more likely to have post-secondary education, a factor associated with improved access to specialist care and greater engagement in preventive health services [39,40,41]. This may have contributed to earlier diagnosis, timely surgical management, and lower observed rates of ovarian cancer in this group.

This descriptive analysis offers a real-world perspective on the relationship between germline *BRCA* mutations and endometriosis, leveraging a large, multicenter, longitudinal cohort. In contrast to previous studies, which were limited by small sample sizes, single-center recruitment, and short follow-up durations, the scale and design of this dataset allowed for a more robust evaluation of endometriosis prevalence and associated risk factors in *BRCA* carriers. Our study included both *BRCA1* and *BRCA2* mutation carriers, allowing for mutation-specific comparisons that remain underexplored in the existing literature. To our knowledge, this is the first study to systematically characterize reproductive, hormonal, and surgical factors associated with endometriosis within a large cohort of *BRCA* mutation carriers, enhancing the generalizability and clinical relevance of our finding.

This study had several limitations, notably that self-reported diagnoses may have led to underreporting or misclassification of endometriosis. Women with undiagnosed or asymptomatic disease may have been included in the comparison group, potentially underestimating differences in ovarian cancer prevalence. Although we did not report on the specific histology of the ovarian cancers, it has been well documented that *BRCA* mutations are associated with the development of serous and endometrioid subtypes [37]. In addition, ethnicity information was not available for this cohort, which is important to acknowledge as endometriosis prevalence varies by race and ethnicity [40]. Given the relatively small number of endometriosis cases and ovarian cancer events, the study was not powered to perform multivariable or time-to-event analyses. A descriptive approach was therefore adopted to characterize patterns and generate hypotheses. Future studies with larger samples, clinically confirmed diagnoses, and tumor histology data are needed to better understand the relationship between endometriosis and cancer risk in *BRCA* mutation carriers.

Overall, our findings suggest that *BRCA* mutations are likely not strongly associated with endometriosis and that endometriosis is unlikely to be a significant risk factor for *BRCA*-ovarian cancer. While traditional risk factors for endometriosis were still present, the influence of early surgical interventions and surveillance practices may help explain these patterns. To our knowledge, this is the first study to describe endometriosis prevalence and associated risk factors within a large cohort of *BRCA* mutation carriers. Future studies should prioritize prospective research to clarify these interactions and their clinical implications, particularly how endometriosis may affect ovarian cancer surveillance and reproductive health in women with a *BRCA* mutation.

## Figures and Tables

**Table 1:** Descriptive characteristics of *BRCA* mutation carriers overall and by endometriosis diagnosis.

Characteristic	Total (n = 16,950)	No Endometriosis (n = 16,547)	Endometriosis ^a^ (n = 403)	*p* ^b^
Year of birth, mean (min–max)	1961 (1903–2004)	1961 (1903–2004)	1962 (1921–1994)	0.40
Age at diagnosis, mean (min–max)	35.2 (13–72)	N/A	35.2 (13–72)	N/A
Follow-up time in years, mean (min–max)	5.5 (2–26)	5.5 (2–26)	8.1 (2–24)	<0.01
Country of residence, n (%)				
USA	5278 (31)	5147 (31)	131 (33)	<0.01
Canada	3743 (22)	3609 (22)	134 (33)	
Poland	5065 (30)	4944 (30)	121 (30)	
Other	2847 (17)	2830 (17)	17 (4)	
Education level, n (%)				
Primary	2548 (33)	2481 (33)	67 (25)	<0.01
Post-secondary	5238 (67)	5033 (67)	205 (75)	
Body Mass Index (BMI) at 18, mean (min–max)	20.9 (10–59)	20.9 (10–59)	20.7 (13–37)	0.32
Age at menarche, mean (min–max)	13.0 (8–30)	13.0 (8–30)	12.7 (9–17)	<0.01
Menopause status, n (%)				
Premenopuase	4651 (28)	4592 (28)	59 (15)	<0.01
Postmenopause	12,255 (72)	11,912 (72)	343 (85)	
Menopause age, mean (min–max)	44.17 (12–73)	44.3 (12–73)	41.5 (23–58)	<0.01
Menopause cause, n (%)				
Natural	2906 (27)	2860 (24)	46 (13)	<0.01
Surgical	6796 (64)	6558 (55)	238 (69)	
Medical	975 (9)	944 (8)	31 (9)	
Parity, n (%)				
Never	3433 (20)	3318 (20)	115 (29)	<0.01
Ever	13,517 (80)	13,229 (80)	288 (71)	
Oral contraceptive, n (%)				
Never	6096 (36)	5995 (36)	101 (25)	<0.01
Ever	10,854 (64)	10,552 (64)	302 (75)	
Postmenopausal HRT ^c^, n (%)				
Never	9722 (79)	9487 (80)	235 (69)	<0.01
Ever	2533 (21)	2425 (20)	108 (31)	
Tubal ligation ^d^, n (%)				
Never	12,984 (88)	12,637 (88)	347 (87)	0.85
Ever	1822 (12)	1772 (12)	50 (13)	
Hysterectomy, n (%)				
Never	11,477 (68)	11,288 (68)	189 (47)	<0.01
Ever	5473 (32)	5259 (32)	214 (53)	
BSO, n (%)				
Never	7794 (46)	7579 (46)	115 (28)	<0.01
Ever	9201 (54)	8906 (53)	295 (72)	

^a^ Women with BSO, hysterectomy and/or ovarian cancer diagnosis before endometriosis diagnosis were excluded. ^b^ The distributions of continuous and categorical variables were compared using the student’s *t*-test and χ^2^ test, respectively. ^c^ HRT use reflects ever use of hormone replacement therapy after surgical or natural menopause. ^d^ Differences between the total cohort size and the sums within individual variables are due to missing responses for specific questionnaire items. N/A= not applicable.

**Table 2:** Distribution of *BRCA* mutation type and ovarian cancer among carriers with and without endometriosis.

Characteristic		No Endometriosis (n = 16,547)	Endometriosis ^a^ (n = 403)	*p* ^b^
Mutation type, n (%)				0.012
Total	16,950			
*BRCA1*	12,169 (72)	11,902 (72)	267 (66)	
*BRCA2*	4781 (28)	4645 (28)	136 (34)	
Ovarian cancer ^c^, n (%)				<0.0001
Total	7794			
Never	6662 (85)	6559 (85)	103 (90)	
Ever	1132 (15)	1120 (15)	12 (10)	

^a^ Women with BSO, hysterectomy and/or ovarian cancer diagnosis before endometriosis diagnosis were excluded. ^b^ The distributions of variables were compared using the χ^2^ test. ^c^ Ovarian cancer reported only in patients with no history of prophylactic BSO.

**Table 3:** Summary of studies investigating the prevalence of endometriosis among *BRCA* mutation carriers.

Study	Country	Population	Study Design	Results
Stratton et al., 1999 [20]	UK	37 women *(BRCA*: n = 11; Non-carriers: n = 26)	Cohort	Endometriosis prevalence was 9.1% (1/11) among *BRCA* mutation carriers and 15.3% (4/26) among non-carriers.
Casey et al., 2013 [21]	USA	174 women with invasive gynecological cancers (*BRCA*: n = 95; *MMR:* n = 79)	Case-only	Endometriosis prevalence was 9.1% (1/11) among *BRCA* mutation carriers and 8.9% (7/79) among women with *MMR* gene mutations. Of the endometriosis cases identified, 81.8% (9/11) were associated with endometrioid carcinomas.
Seidman and Krishnan, 2016 [22]	USA	403 women (*BRCA*: n = 38; High-risk without mutation: n = 79; low-risk without mutation: n = 286)	Cohort	Endometriosis prevalence was 34.2% (13/38) among *BRCA* mutation carriers, 30.4% (24/79) in high-risk individuals without a mutation, and 18% (51/286) in non-high-risk individuals. The overall prevalence of endometriosis was 22% (88/403).
Thompson et al., 2018 [23]	IRL	130 women (*BRCA:* n = 46, Non-Carriers: n = 19, Untested: n = 65)	Cohort	Endometriosis prevalence was 2.2% (1/46) among *BRCA* mutation carriers and 7.1% (6/84) in non-carriers or untested individuals.
Grandi et al., 2020 [24]	ITA	116 women with *BRCA* mutations (Previous RRSO: n = 25; Actual RRSO: n = 29; No RRSO: n = 62)	Cohort	The overall prevalence of endometriosis was 4.3% (5/116).
Gersekowski et al., 2024 [25]	AUS	4926 women with ovarian cancer (*BRCA:* n = 637; Non-carriers n = 4289) * Data on endometriosis was missing for 1648 women	Pooled Case-only	Endometriosis prevalence was 8.6% (41/476) among *BRCA* mutation carriers and 10.2% (285/2802) among non-carriers The overall prevalence of endometriosis was 9.9% (326/3278) The positive association between endometriosis and ovarian cancer risk was weaker for *BRCA* carriers compared to non-carriers (IRR = 0.75, 95% CI: 0.54–1.01)
Loizzi et al., 2024 [26]	ITA	184 women with *BRCA* mutations (Personal history of cancer: n = 14)	Cohort	Endometriosis was reported in 1.6% (3/184) of patients, all of whom had no personal or family history of cancer.

* NS = Not statistically significant, numerical data not reported; N/A = Not available; RRSO = risk-reducing salpingo-oophorectomy; OR = Odds ratio; IRR = interaction risk ratio; CI = confidence interval.

## Data Availability

The data presented in this study are available on reasonable request from the corresponding author. The data are not publicly available due to institutional privacy policies.
